# A Novel Hypoxic-Angiogenesis-Immune-Related Gene Model for Prognostic and Therapeutic Effect Prediction in Hepatocellular Carcinoma Patients

**DOI:** 10.1155/2022/9428660

**Published:** 2022-01-12

**Authors:** Wen Lv, Qi Yao

**Affiliations:** ^1^Emergency Department, Shenzhen People's Hospital, Shenzhen 518020, China; ^2^Anorectal Surgery, Shenzhen People's Hospital, Shenzhen 518020, China

## Abstract

**Background:**

Hepatocellular carcinoma (HCC) is one of the most heterogeneous malignant tumors that have been discovered so far, which makes the prognostic prediction difficult. The hypoxia, angiogenesis, and immunity-related genes (HAIRGs) are closely related to the development of liver cancer. However, the prognostic and treatment effect of hypoxia, angiogenesis, and immunity-related genes in HCC continues to be further clarified.

**Methods:**

The gene expression quantification data and clinical information in patients with liver cancer were downloaded from the TCGA database, and HAIRG signature was built by using the least absolute shrinkage and selection operator (LASSO) technique. Patient from the ICGC database validated the model. Then, tumor immune dysfunction and exclusion (TIDE) algorithm was applied to estimate the clinical response to immunotherapy and the sensitivity of drugs was evaluated by the half-maximal inhibitory concentration (IC_50_).

**Result:**

The HAIRGs were identified between the HCC patients and normal patients in the TCGA database. In univariate Cox regression analysis, seventeen differentially expressed genes (DEGs) were associated with overall survival (OS). An eight HAIRG signature model was constructed and was used to divide the patients into two groups according to the median value of the risk score base on the TCGA dataset. Patients in the high-risk group had a significant reduction in OS compared to those in the low-risk group (*P* < 0.001 in the TCGA, *P* < 0.001 in the ICGC). For TCGA and ICGC databases of univariate Cox regression analyses, the risk score was used as an independent predictor of OS (HR > 1, *P* < 0.001). Functional analysis showed that the relevant immune pathways and immune responses were enriched, cellular component analysis showed that the immunoglobulin complex and other related substances were enriched, and immune status existed a difference in the high- and low-risk groups. Then, the tumor immune dysfunction and exclusion (TIDE) algorithm presented differences in immune response in the high- and low-risk groups (*P* < 0.05), and based on drug sensitivity prediction, patients in the high-risk group were more sensitive to cisplatin compared to those in the low-risk group in both the TCGA and ICGC cohorts (*P* < 0.05).

**Conclusions:**

HAIRG signature can be utilized for prognostic prediction in HCC, while it can be considered a prediction model for clinical evaluation of immunotherapy response and chemotherapy sensitivity in HCC.

## 1. Introduction

Liver cancer is the sixth most commonly diagnosed cancer in terms of morbidity and the fourth leading cause of cancer related to death [1]. According to the WHO prediction, more than one million people will die of liver cancer in 2030, based on an annual projection [1]. Factors such as chronic hepatitis B or C, alcohol addiction, metabolic liver disease (particularly NAFLD), and dietary toxicosis, for instance aflatoxin and aristolochic acid increase the risk of liver cancer development [2]. Being a highly heterogenous form of liver cancer, hepatocellular carcinoma (HCC) generates a low survival rate and increases the difficulty of overall survival prediction for HCC patients. Therefore, developing a precise prognostic and therapeutic model will help to clinical evaluation and prolong the survival time for HCC patients.

Tumor microenvironment (TME) can be affected by some important components, such as hypoxia, angiogenesis, and immune cells, which can influence tumor growth [3]. For the development of HCC, hypoxic stimulation can cause bone marrow cells enter to the TME; then, they differentiate into tumor-associated macrophages or neutrocytes and secrete cytokines and proangiogenic growth factors to promote tumor development [4]. Meanwhile, hypoxia can stimulate the release of hypoxia-inducible factors (HIF), which signal both natural immune cells and HCC cells. It is beneficial to the recruitment and maintenance of prototumor immune cells, inhibition of antitumor immune cells, and promotion of immune escape [5]. Immune escape plays an important role in the occurrence and metastasis of hepatic carcinoma [6]. In a hypoxic environment, some suppressive immune cells, like regulatory T cells and M2 macrophages, are frequently recruited to cancer tissues to form the immunosuppressive microenvironment in HCC, which can secret some procancer inflammatory cytokines and activate the STAT3 and NF-*κ*B signaling pathways [6]. Therefore, hypoxia, angiogenesis, and immune response act as critical roles in the progression of HCC. Nevertheless, it is still unclear whether hypoxia, angiogenesis, and immunity-related genes (HAIRGs) were correlated with the prognosis and immune checkpoint therapy response.

In our study, we first established a multigene prognostic model base on HAIRGs in the TCGA database and validated it in the ICGC database. Finally, we conducted functional enrichment analysis and immune response prediction to explore the underlining mechanism of the prognostic model.

## 2. Materials and Methods

### 2.1. Collecting the Data

Quantitative gene expression data and clinical information of liver cancer patients were downloaded from the TCGA database (https://portal.gdc.cancer; containing 374 liver cancer samples and 50 normal tissue samples) and ICGC database (http://www.ncbi.nlm.nih.gov/geo/; containing 231 liver cancer samples). The genetic information about hypoxia, angiogenesis, and immunity was downloaded from the GeneCards database (https://www.genecards.org/). The TCGA and ICGC databases were used for training queue and validation queue, respectively.

### 2.2. Screening and Identifying HAIRG Signature Associated with LC Prognosis

The HAIRGs were matched (the top 500 genes of the three gene sets). DEGs between hypoxia, angiogenesis, and immunity-related genes were recognized by “limma” R package with the error discovery rate of <0.05. The overlapping prognostic DEGs were incorporated into the LASSO Cox regression using the “glmnet” R package. Univariate Cox analysis was accomplished for OS using the “survival” R package to screen HAIRGs with prognostic potential. According to the minimum criteria, the tenfold cross-validation was used in the penalty parameter (*λ*). Based on the expression of each gene and the corresponding regression coefficient, a risk score was calculated for each patient. The formula: Risk score = SUM (expression of each gene × corresponding coefficient). Patients with liver cancer were divided into the high- and low-risk groups according to the median value of the risk score. Principal components analysis (PCA) was carried out by using the “prcomp” function of the “stats” R package based on signature gene expression in the the TCGA database. In addition, t-SNE was implemented through the “Rtsne” R package to investigate the distribution of the two groups.

### 2.3. The Predictive Nomogram Construction and Evaluation

Univariate and multivariate Cox regression analyses were executed to determine whether the risk score was an independent prognostic predictor for OS compared to other clinical features in the TCGA database. The “rms” R package was utilized to construct a predictive nomogram and corresponding calibration maps based on independent predictive factors. To evaluate the predictive power of the nomogram, a time-dependent receiver operating characteristic (ROC) curve analysis was performed by using the “time ROC” R package. Patients from ICGC were analyzed by using the same formula as that for the TCGA database. The sensitivity and specificity of the nomogram were tested by the ROC curves.

### 2.4. Functional Enrichment Analysis and Immunotherapy Response Predictions

Gene Ontology (GO) and Kyoto Encyclopedia of Genes and Genomes (KEGG) analyses based on the DEGs were analyzed by using the STRING database (∣log2FC | ≥1, FDR < 0.05) in the high- and low-risk groups. The *P* value was regulated by the BH method. The single-sample gene set enrichment analysis (ssGSEA) was utilized to calculate the infiltrating score of 16 immune cells and the activity of 13 immune-related pathways [7] in the “gsva” R package.

### 2.5. Immunotherapy Response Predictions for HCC Patients

TIDE (http://tide.dfci.harvard.edu/) is a combination model of T cell dysfunction and exclusive expression characteristic that can calculate and simulate the tumor immune escape [8]. The TIDE algorithm was utilized for predicting the clinical response to immunotherapy in the TCGA and ICGC cohorts.

### 2.6. Evaluation of the Sensitivity of Drugs

The Genomics of Drug Sensitivity in Cancer (GDSC; https://www.cancerrxgene.org/) database was used to assess the sensitivity of chemotherapy drugs [9]. The half-maximal inhibitory concentration (IC50) was assessed by using the pRRophetic package in R.

### 2.7. Statistical Analysis of the Data

All statistical analyses were processed by the R software (version 4.0.3). Student's two-sided *t*-test was used to compare the expression of genes in HCC tissues and nearby nontumorous tissues. The Kaplan-Meier analysis and log-rank test were used to compare the difference of OS among groups. Univariate and multivariate Cox regression analyses were used to determine independent predictors of OS. SsGSEA score of immune pathways or cells were compared between the two groups by Mann–Whitney test. *P* value < 0.05 were considered statistically significant if no specific requirement is made, and all *P* values were double-tailed. Since the TCGA, ICGC, and GeneCards databases are publicly available, this study strictly followed access policies for databases and publication guidelines; thus, ethical approval from a local ethics committee was not required.

## 3. Results

### 3.1. Identification of Prognostic HAIRGs in the TCGA Cohort

**Figure 1:**
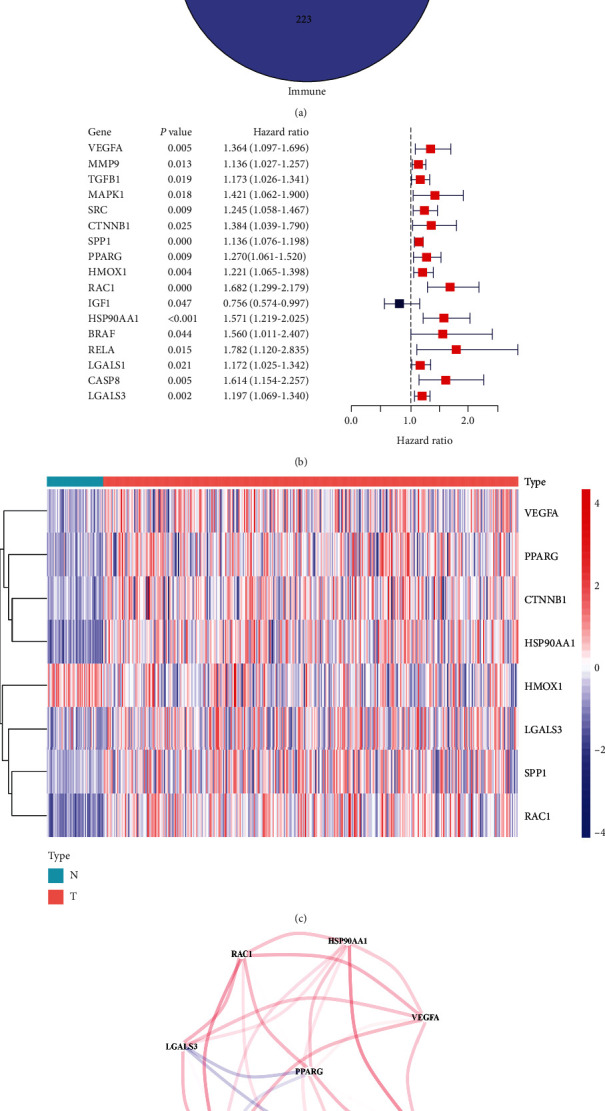
Identification of the hypoxia, angiogenesis, and immune-associated genes in the TCGA queue. (a) The Venn diagram to recognize DEGs between the hypoxia, angiogenesis, and immune genes that were associated with OS. (b) Forest plots showing the results about gene expression and OS using univariate Cox regression analysis. (c) The 7 overlapping genes were all upregulated, and one gene was downregulated in tumor tissue. (d) The correlation network of genes that had been selected. The correlation coefficients are showed in different colors.

All 44 DEGs between hypoxia, angiogenesis, and immune-related genes were identified (Figure 1(a)). Univariate Cox regression analysis showed 17 HAIRGs (VEGFA, MMP9, TGFB1, MAPK1, SRC, CTNNB1, SPP1, PPARG, HMOX1, RAC1, IGF1, HSP90AA1, BRAF, RELA, LGALS1, CASP8, and LGALS3) were significantly related to the OS for HCC patients, sixteen of which were high hazard ratio genes (HR > 1) and one was protective genes (HR < 1) (Figure 1(b)). Then, we picked out 8 HAIRGs (VEGFA, CTNNB1, PPARG, HSP90AA1, HMOX1, LGALS3, SPP1, and RAC1) from the 17 HAIRG model through the LASSO Cox regression, upregulated genes accounted for 7/8 in tumor tissue, which was shown by a heat map (Figure 1(c)). The correlation between above the 8 HAIRGs was shown in Figure 1(d).

### 3.2. Building a Prognostic Model in the TCGA Cohort

**Figure 2:**
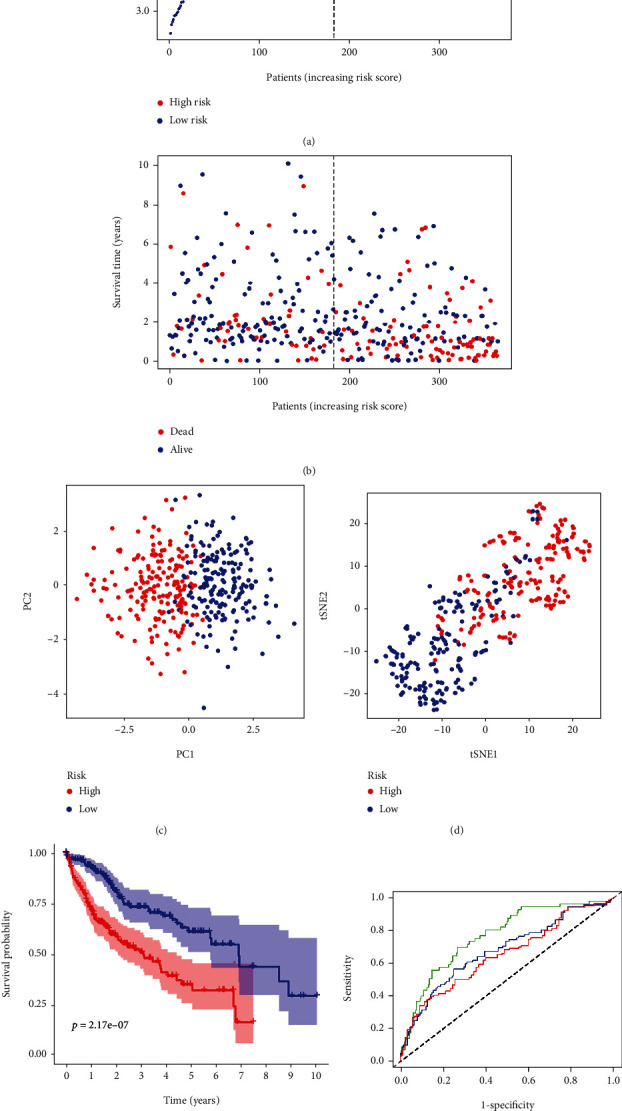
Prognostic analysis of the characteristic models of 8 genes in the TCGA cohort. (a) The distribution and median value of the risk score in the TCGA cohort. (b) The distributions of OS condition, OS, and risk score in the TCGA queue. (e) Kaplan-Meier curves of OS in the high- and low-risk TCGA cohorts for HCC patients. (c) PC plot of the TCGA cohort. (d) t-SNE analysis of the TCGA cohort. (f) The area under the curve of time-dependent ROC curves validated the prognostic manifestation of the risk score in the TCGA.

Based on LASSO Cox regression analysis, eight genes of HAIRG prognostic model related to OS in patients with HCC were constructed (VEGFA, CTNNB1, SPP1, PPARG, HMOX1, RAC1, HSP90AA1, and LGALS3). They were subjected to construct a hypoxia, angiogenesis, and immune-related prognostic model by using the following formula: Risk score = SUM (0.097∗expression of VEGFA + 0.093∗expression of CTNNB1 + 0.070∗expression of SPP1 + 0.129∗expression of PPARG + 0.021∗expression of HMOX1 + 0.117∗expression of RAC1 + 0.151∗expression of HSP90AA1 + 0.060∗expression of LGALS3). Utilizing median value of the risk score, the patients were classified into the high-risk group (*n* = 212) and the low-risk group (*n* = 212) (Figure 2(a)). From Figure 2(b), we could see that patients in the high-risk group were likely to have a shorter lifespan than those in the low-risk group (Figure 2(e)). Through PC and T-SNE analyses, the two subgroups showed the discrete distribution (Figures 2(c) and 2(d)). In addition, AUC values of 1, 2, and 3 years for the 8-gene signature were 0.771, 0.681, and 0.654 by ROC analysis (Figure 2(f)).

### 3.3. Verifying the HAIRG Signature in the ICGC Database

**Figure 3:**
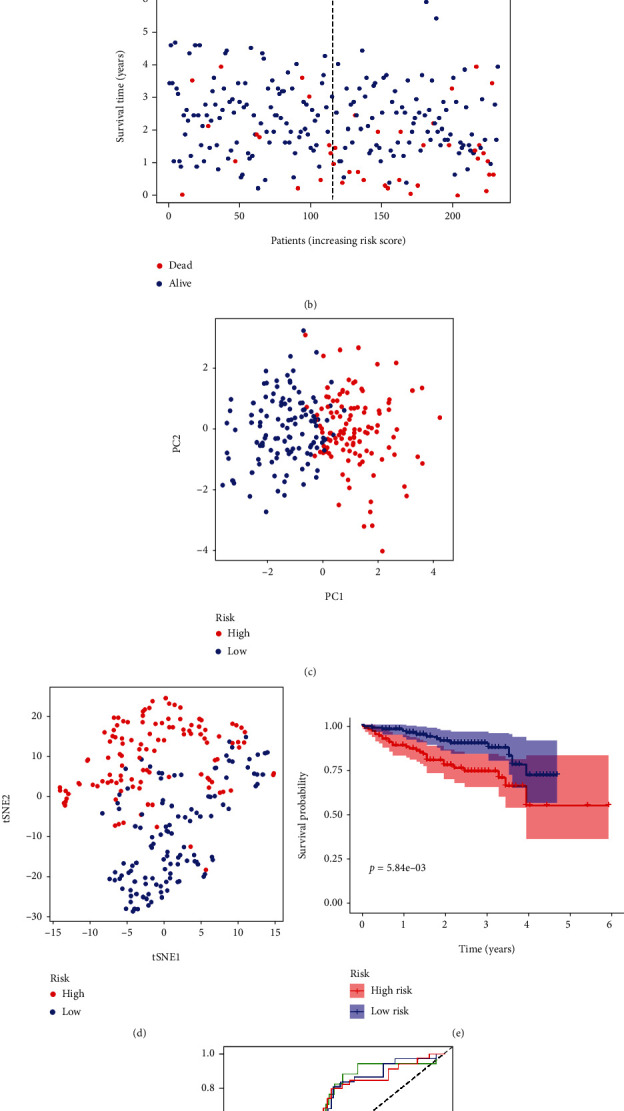
8-gene characteristic model prognostic analyzed in ICGC cohort. (a) The distribution and median value of the risk score in the ICGC cohort. (b) The distributions of OS condition, OS, and risk score in the ICGC queue. (e) Kaplan-Meier curves of OS in the high- and low-risk TCGA cohorts for HCC patients in the ICGC cohort. (c) PC plot of the ICGC cohort. (d) t-SNE analysis of the ICGC cohort. (f) The area under the curve of time-dependent ROC curves validated the prognostic manifestation of the risk score in the ICGC queue.

To examine the stability of the model established in the TCGA database, we used the same formula as the TCGA database to calculate the risk score for each patient in the ICGC database. Similar to the TCGA database, patients were also classified into high-risk group (*n* = 115) or low-risk group (*n* = 116) in the ICGC according to the median value of the risk score (Figure 3(a)). The results were similar to the TCGA cohort, PC and T-SNE analyses verified the reliable aggregation ability of risk score in ICGC database (Figures 3(c) and 3(d)). It was known that patients in the high-risk group were likely to die earlier and had a shorter lifespan compare to those in the low-risk group (Figures 3(b)–3(e)). Besides, after completing the ROC analysis, AUC values of 1, 2, and 3 years were 0.654, 0.673, and 0.657, respectively (Figure 3(f)).

### 3.4. Independent Prognostic Value of the HAIRG Signature

**Figure 4:**
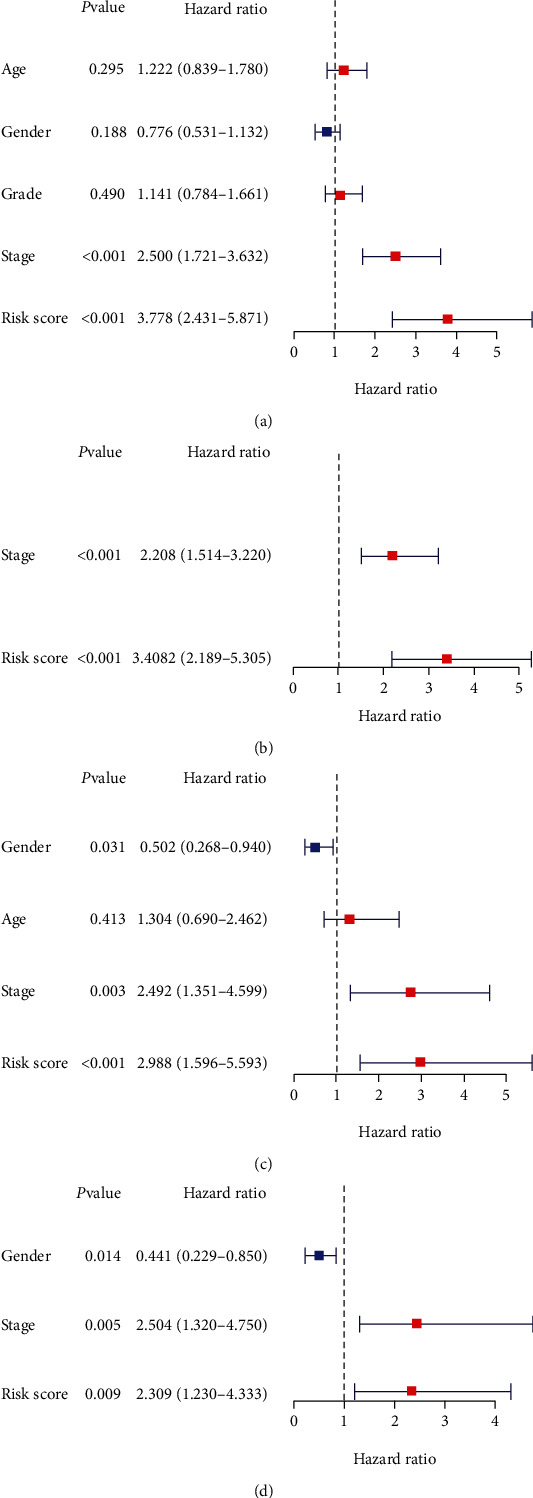
Results of the univariate Cox regression analyses about OS. (a, b) Univariate and multivariate Cox regression analyses about OS in the TCGA cohort. (c, d) Univariate and multivariate Cox regression analyses about OS in the ICGC cohort.

Univariate and multivariate Cox regression analyses were performed to determine whether risk score was an independent prognostic predictor of OS. Univariate Cox regression analysis showed there was a significant correlation between risk score and OS in the TCGA database (HR = 3.778, 95%CI = 2.431-5.871, *P* < 0.001, Figure 4(a)) and in the ICGC database (HR = 2.988, 95%CI = 1.296-5.593, *P* < 0.001, Figure 4(c)). Multivariate Cox regression analysis showed there was a meaningful relationship between the risk score and OS in the TCGA database (HR = 3.408, 95%CI = 2.189-5.305, *P* < 0.001, Figure 4(b)) and in the ICGC database (HR = 2.309, 95%CI = 1.230-4.333, *P* = 0.009, Figure 4(d)).

### 3.5. Establishment and Validation of Nomogram

**Figure 5:**
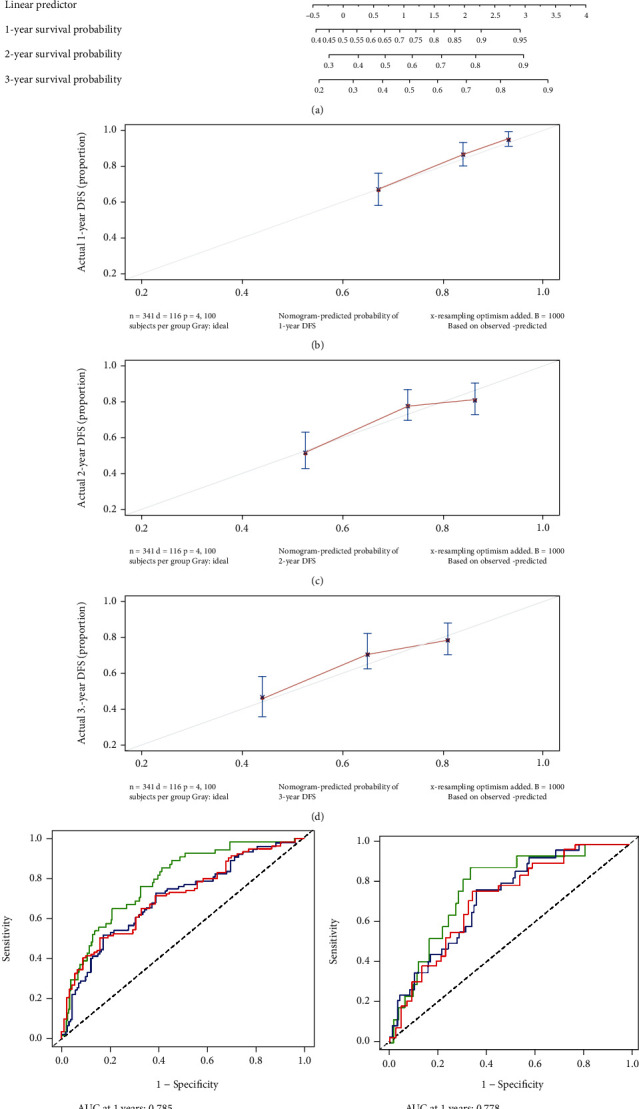
(a) Prognostic nomogram for liver cancer patients. (b–d) OS prediction at 1, 2, and 3 years by the corrected curve of the nomogram. (e, f) Area under the curve of time-dependent ROC curves in the ICGC cohort. Time-dependent ROC for 1-year, 2-year, and 3-year OS of the nomogram in the TCGA (e) and in the ICGC (f).

Univariable Cox regression analysis showed the higher cancer stage and risk scores were independent risk factors for prognosis (*P* < 0.05). Based on the multivariate analysis, tumor stage and risk score were also served as independent predictors for OS (*P* < 0.05). Later, these two variables were utilized to build a nomogram of OS, including the tumor stage and risk score (Figure 5(a)). The scores for each variable were added up, and the total was projected to the bottom of the scale, making it easy to calculate estimated 1-, 2- and 3-year probabilities of OS. The time-dependent ROC curves and calibration curves were performed to clear and definite the discriminant advantages of nomogram. The AUC values of the 1-, 2-, and 3-year nomogram for OS in the TCGA cohort were 0.785, 0.700, and 0.708 (Figure 5(e)). In the ICGC database, the AUC values of 1-, 2-, and 3-year nomogram for OS were 0.778, 0.732, and 0.722, respectively (Figure 5(f)).

### 3.6. Functional Analysis in the TCGA and ICGC Queues

**Figure 6:**
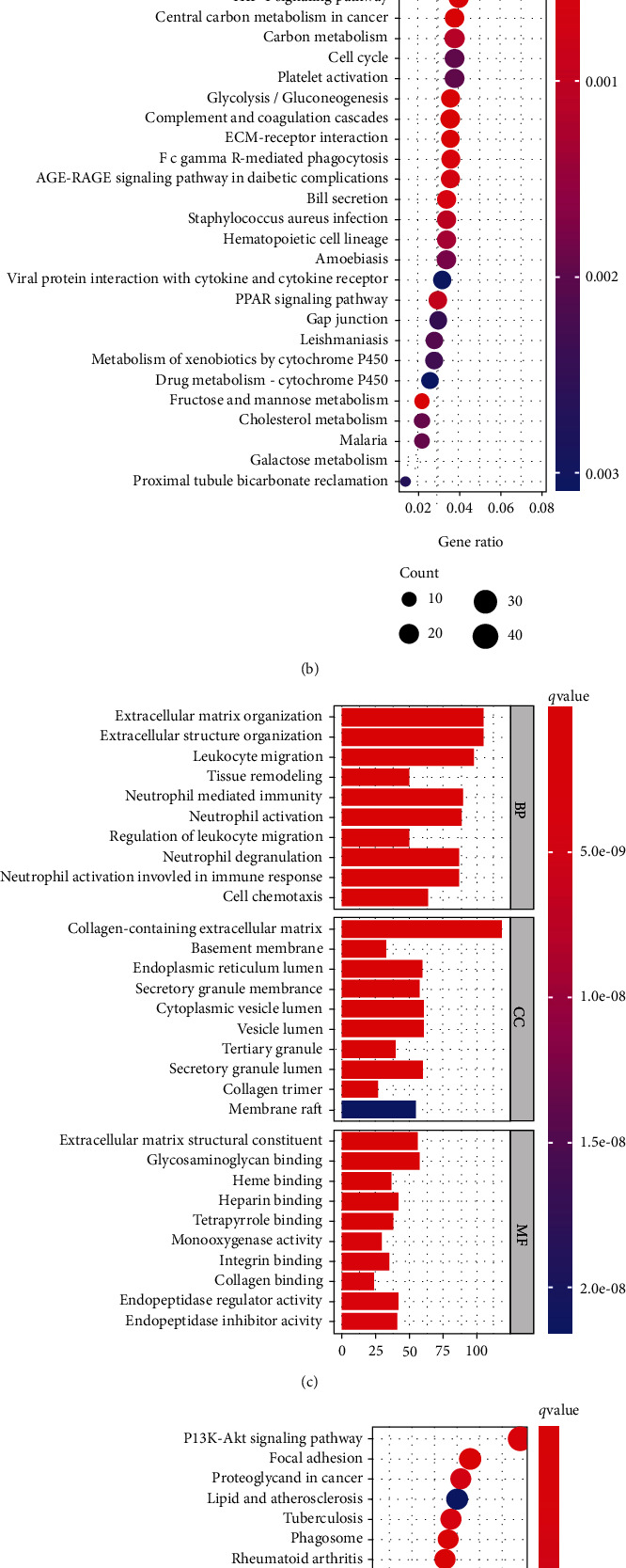
Biological process (BP), cellular component (CC), and molecular function (MF) analysis between different riskscore genes. (a, b) Typical results of KEGG analyses (a, c) and GO (b, d) in the TCGA cohort. (c, d) Results of KEGG analyses and GO in the ICGC cohort. The pink rectangles represent the overlap of immune-related pathways in the TCGA and ICGC cohorts.

To explore the potential molecular mechanism of the signature, we used the DEGs to perform GO enrichment and KEGG pathway analyses in the high- and low-risk groups. As we expected, the DEGs were significantly enriched in many immune-related biological processes, such as phagocytosis and leukocyte migration in the TCGA cohort (Figure 6(a)). DEGs were also gathered in a few hypoxia and immune-related molecular functions in the TCGA and ICGC databases, for instance, heme binding and antigen binding (*P* < 0.05, Figures 6(a) and 6(c)). For KEGG analysis, the differentially expressed HAIRGs were gathered in essential pathways associated with immune and cancer progression, for example, proteoglycans in cancer, phagosome, complement and coagulation, and PI2K-Akt signaling pathway (*P* < 0.05, Figures 6(b) and 6(d)).

**Figure 7:**
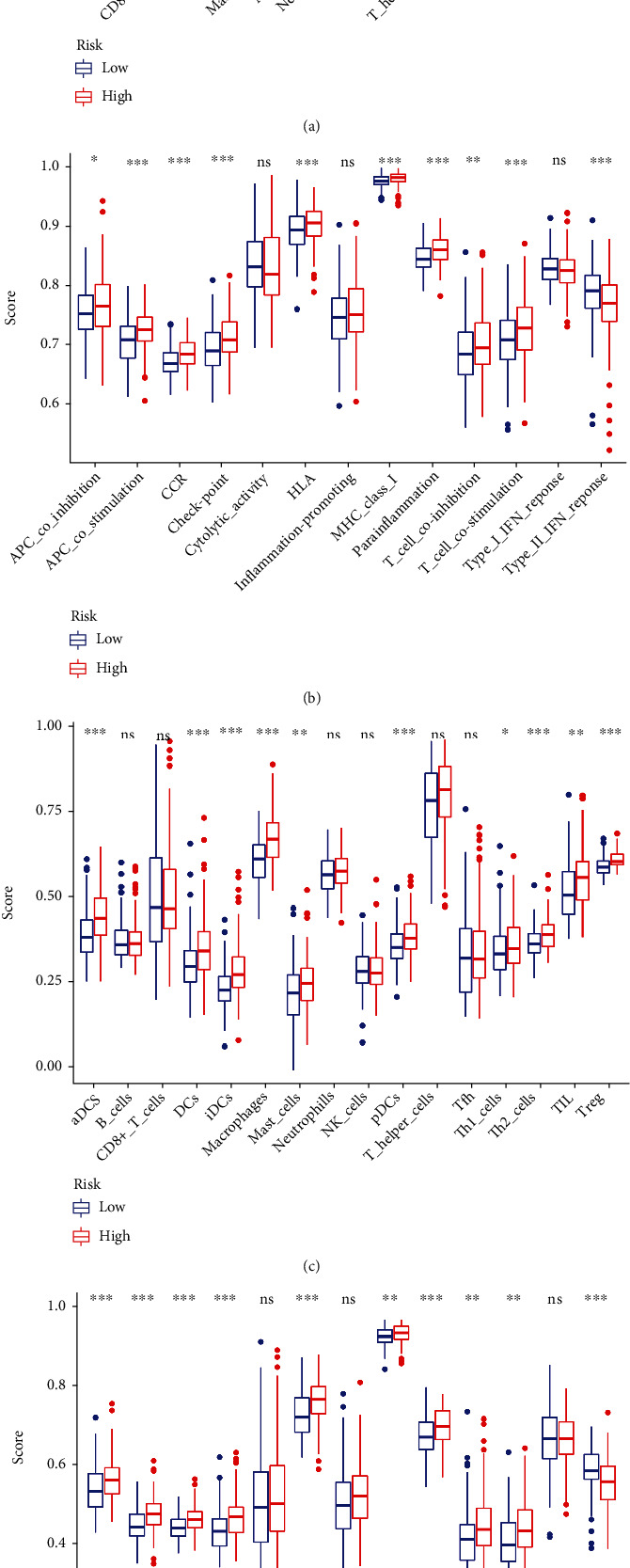
Comparing the ssGSEA scores in high- and low-risk groups in the TCGA cohort and ICGC cohort. (a, b) The scores of sixteen immune cells and thirteen immune-related functions are showed in boxplots in TCGA cohort. (c, d) Results of ssGSEA scores in the ICGC cohort. Adjusted *P* values were presented, as follow: ns: not significant; ^∗^*P* < 0.05, ^∗∗^*P* < 0.01, and ^∗∗∗^*P* < 0.001.

We quantified the enrichment score of different immune cell subsets, associated functions or pathways using ssGSEA to further investigate the correlation between risk score and immune status. In the TCGA cohort, there were significant differences in the antigen recognition process (including the score of T cell coinhibition, HLA, APC coinhibition, IDCs, and ADCs) between the high-risk group and the low-risk group (Figures 7(a) and 7(b)). The immunobiological processes in the GO analyses scored higher in the high-risk group in the TCGA cohort (*P* < 0.05, Figure 6(a)). However, scores of the type I IFN response, type II IFN response, NK cells, and mast cells were lower in the high-risk group, compared to that in the low-risk group. In contrast, scores of macrophages, Treg cell, TIL, and HLA were higher in the high-risk group (*P* < 0.05, Figures 7(a) and 7(b)). The differences of aDCs, DCs, iDCs, pDCs, Th2 cells, Treg, APC costimulation or inhibition, checkpoint, and type II IFN response between the two groups through comparisons in the ICGC database (*P* < 0.05, Figures 7(c) and 7(d)). In the TCGA and ICGC cohorts, the macrophage and ADC score existed the greatest statistical difference in the high- and low-risk groups, which were also consistent with the results from the GO analysis.

### 3.7. Prediction of Immunotherapeutic Response and Immune Checkpoint Expression Pattern in HCC Patients

**Figure 8:**
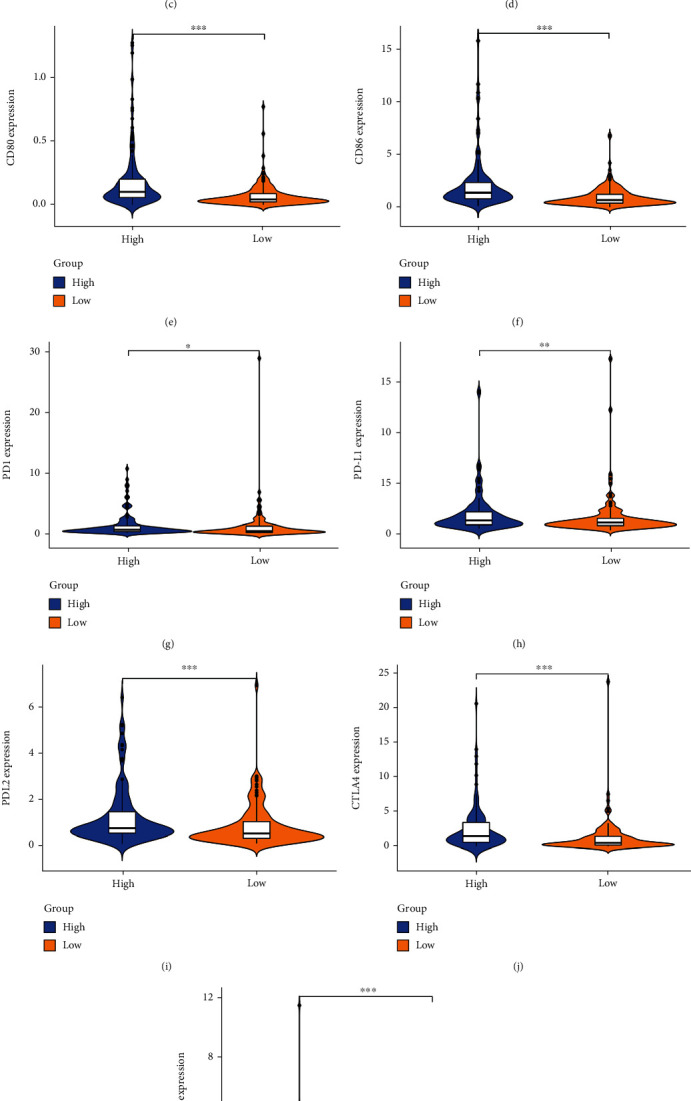
The expression patterns of immune checkpoint for HCC patients. (a–f) There are six major immune checkpoint molecules expressed in the violin plots, namely, PD1, PDL1, PDL2, CTLA4, CD80, and CD86, in the TCGA cohort. (g–l) correlation between HAIRGs risk score and PD1, PDL1, PDL2, CTLA4, CD80, and CD86 expressions in the ICGC cohort.

There are six main immune checkpoints PD1, PDL1, PDL2, CTLA4, CD80, and CD86; their expression level of HCC were detected and compared in the TCGA and ICGC databases. All six immune checkpoints were over expression in the high-risk group in the TCGA database (Figures 8(a)–8(f)). Besides, the six immune checkpoints, PD1, PDL1, PDL2, CTLA4, CD80, and CD86, were also over expression in the high-risk group in the ICGC database (Figures 8(g)–8(l)). So, the expression of immune checkpoint PD1, PDL1, PDL2, CTLA4, CD80, and CD86 in the high-risk group was significantly higher than the one in the low-risk group.

**Figure 9:**
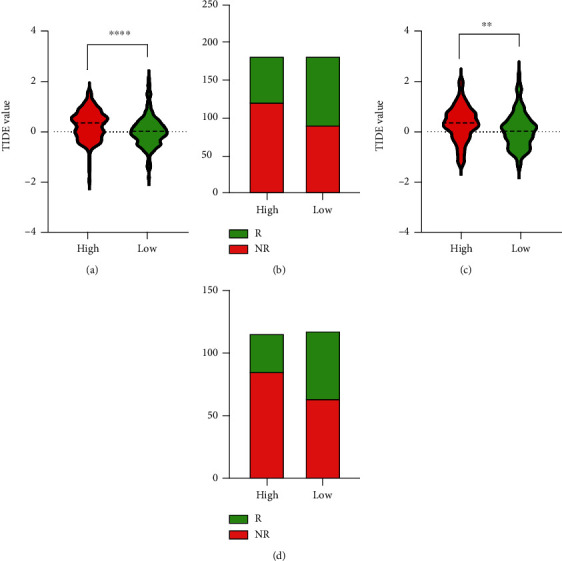
Prediction of reaction to immunotherapy in patients with HCC. The violin plots show the distribution of the TIDE value in immunotherapy response between the TCGA (a) and ICGC cohort (c). The proportion of patients with response/no response to immunotherapy based on the two risk stratifications, in TCGA cohort (b) and in ICGC cohort (d).

Later, the possibility of predicting immunotherapeutic responses is based on the tumor immune dysfunction and rejection (TIDE) algorithm in the TCGA-ICGC cohort. Then, we found that high TIDE value was related to the high-risk group, while low TIDE value was related to the low-risk group in the TCGA database (*P* < 0.05, Figure 9(a)) and ICGC cohort (*P* < 0.05, Figure 9(c)). The proportion of immunotherapy response was significantly higher among patients in the low-risk group than the patients in the high-risk group in TCGA (*P* < 0.05, Figure 9(b)) and ICGC cohort (*P* < 0.05, Figure 9(d)), which indicated that immunotherapy has a better response rate on the low-risk group.

### 3.8. Sensitivity of Chemotherapy Drugs between TCGA and ICGC Cohorts

**Figure 10:**
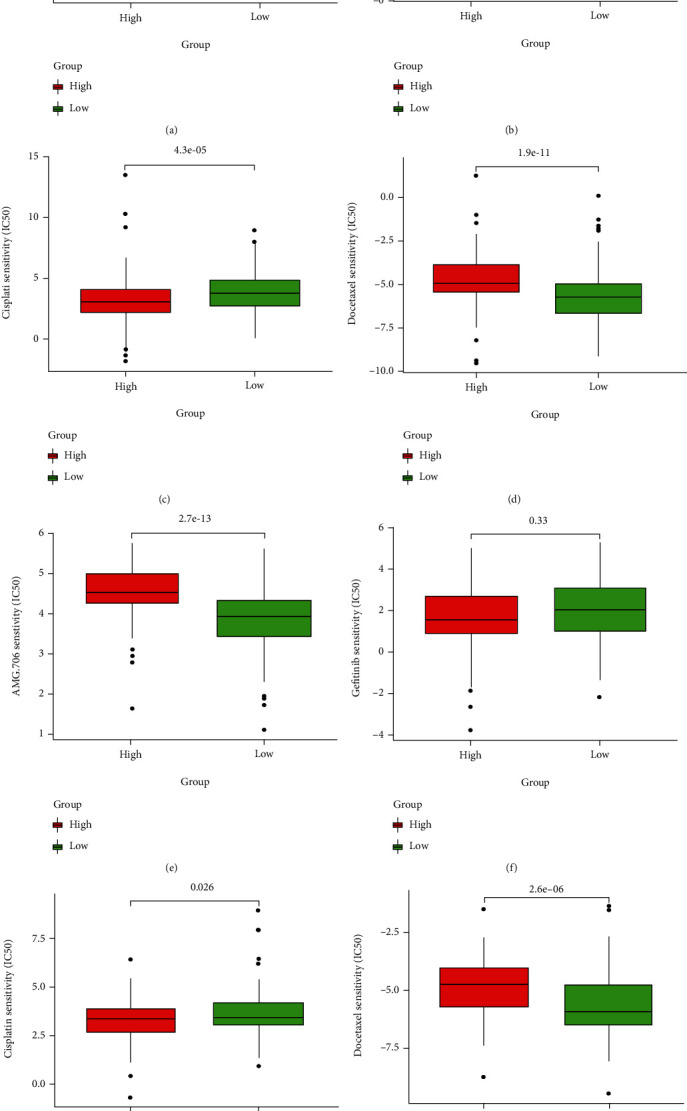
Boxplots present the estimated IC50 value of chemotherapy drugs in two risk groups. (a) AMG 706, (b) gefitinib, (c) cisplatin, and (d) docetaxel in the TCGA. (e) AMG 706, (f) gefitinib, (g) cisplatin, and (h) docetaxel in the ICGC.

Adjuvant chemotherapy is an alternative treatment of HCC. Thus, it is important to predict the sensitivity of chemotherapy, which can help clinicians to choose the best chemotherapy regimen. The calculated IC_50_ levels of Cisplatin (*P* = 4.3*e* − 05) were lowered in the high-risk group than in the low-risk group. AMG 706 (*P* = 3.8*e* − 16), gefitinib (*P* = 2.3*e* − 05) and docetaxel (*P* = 1.9*e* − 11) was lowered in the low-risk group in the TCGA database (Figures 10(a)–10(d)), indicating the high-risk group was more susceptible to cisplatin while the low-risk group was more sensitive to AMG 706, gefitinib, and docetaxel drugs. The estimated IC_50_ levels of cisplatin (*P* = 0.026) were lowered in the high-risk group, but AMG 706 (*P* = 2.7*e* − 13) and docetaxel (*P* = 2.6*e* − 06) was lowered in the low-risk group in the ICGC database (Figures 10(e)–10(h)). In brief, the high-risk group was more susceptible to cisplatin, but the low-risk group was more susceptible to AMG 706 and docetaxel.

## 4. Discussion

In our study, 17 HAIRGs were firstly identified to be related to overall survive in HCC patients. Then, basing on the LASSO Cox regression analysis, 8-gene of prognostic model of HAIRGs related to OS in patients with HCC was constructed, which is based on the LASSO Cox regression analysis. At the same time, the model was validated in the ICGC cohort. In the present study, we used GO and KEGG to analyze the differentially expressed HAIRGs between liver cancer patients and normal controls. The GO results showed differentially expressed HAIRGs were significantly enriched in phagocytosis, leukocyte migration, immune response-activating cell surface receptor signaling pathway, neutrophil activation, heme binding, and antigen binding (in terms of BP and MF). These results suggested that differentially expressions of HAIRGS were related to the development of liver cancer. Moreover, for KEGG analysis, it showed differentially expressed HAIRGs were involved in the development of liver cancer, which also indicated HAIRGs may regard as a potential biomarker for HCC. Then, immunotherapy response predication exhibited that the low-risk group was associated with low TIDE score and had a better immunotherapy effect in the TCGA and ICGC cohorts. Furthermore, for drug sensitivity analysis in HCC patients, we found that the high-risk group was more susceptive to cisplatin, compared to the low-risk group in the TCGA and ICGC cohorts. Therefore, our result firstly provides a HAIRG signature model which can be utilized for prognostic, immunotherapy response, and chemotherapy sensitivity prediction in HCC.

Currently, the most commonly use of the method for predicting liver cancer is Okuda System Staging. However, this model has some limitations. Over time, the early diagnosis of HCC has changed due to improved diagnostic methods. At the same time, Okuda staging is insufficient to stratify patients prior to radical or palliative treatment [10]. A new and reliable prognostic model is an urgent needed to predict OS in HCC patients. In our study, we found the potential role of the HAIRGs in HCC patients and the possibility of constructing a prognostic model with these HAIRGs. Besides, the 3-year predictive ability of the nomogram for OS was 0.708 in the TCGA cohort and 0.722 in the ICGC database for the model.

The prognostic model consisted of HAIRGs in the present study, including VEGFA, CTNNB1, SPP1, PPARG, HMOX1, RAC1, HSP90AA1, and LGALS3. VEGFA, also called vascular endothelial growth factor-A, is not the only, major factor driving tumor vascular bed dilation [11]. Angiogenesis in HCC depends mainly on VEGFA-driven response, which leads to vascular system dysfunction to a large extent. The reason for this is not clear, although it seems that some aspects of the angiogenic environment stimulated by VEGFA-stimulated angiogenic milieu (high levels of microvascular permeability and density) are capable of promoting tumor expansion [11]. CTNNB1 may play a vital role in metabolic reprogramming and cell proliferation in HCC. Phosphorylation sites associated with CTNNB1 mutations were confirmed on key metabolic enzymes, including ALDOA, and the function of phosphate-ALDOA about promoting metabolic reprogramming and cell proliferation was demonstrated [12]. Secreted phosphoprotein-1 (SPP1) is a secreted arginine-glycine-aspartate (RGD) containing phosphoprotein [13]. SPP1 may play a role as a miR-181c-targeted growth promoter of HCC [14]. PPARG had three subtypes, namely, PPARG1, PPARG2, and PPARG3 [15]. The susceptibility to HCC may be affected by PPARG gene polymorphism [16]. The expression of PPARG was upregulated in lung, prostate, colorectal, bladder, and breast tumors [17]. Heme oxygenase-1 (HMOX-1), an important catalytic enzyme in heme degradation, is increased under stressful conditions [18]. The elevated expression of HMOX-1 in a variety of malignant tumors is related to the tumor microenvironment resistance to tumor cell growth, angiogenesis, metastasis, chemotherapy, and radiotherapy [19]. It suggested that HMOX-1 is a protective gene in liver cancer. HSP90AA1 had the vital functions in the process of the assembly, manipulation, folding, and degradation of its customer proteins [20]. It had been shown to be overexpressed in multifarious human cancer, including liver, breast, endometrial, ovarian, colon, lung, and prostate cancers [21–24]. LGALS3 (galectin-3) is a multifunctional protein, which has a variety of biological functions, including tumor cells proliferation and differentiation, angiogenesis, tumor progression, and metastasis [25]. Galectin-3 is associated with a lot of cancers, such as mesothelioma, breast, HCC, and colon cancers [26–28]. In summary, seven of the genes (VEGFA, CTNNB1, SPP1, PPARG, RAC1, HSP90AA1, and LGALS3) were highly unregulated in patients with liver cancer. Only one gene (HMOX-1) was downregulated, hinting which may be a protective gene for HCC. Whether these genes affect the prognosis of HCC patients by influencing hypoxia, angiogenesis, and immune processes remained to be elucidated.

In functional analysis, we found that different HAIRG signatures of HCC shown a significantly distinguished immune microenvironments, such as immune infiltration levels which including aDCs, DCs, iDCs, pDCs, Th2 cells, and Treg. Moreover, the functional differences that T cell costimulation or inhibition, checkpoint, and type II IFN response were also found in the different groups of HAIRG signature in HCC. The IFN pathway was closely related to the progression of HCC patients. The interferons are divided into three types, type I (IFN-*α* and IFN-*β*), type II (IFN-*γ*), and type III. Currently, it is known that interferon- (IFN-) *α* is one of the vital treatment options for patients with liver cancer [29]. IFN-*α* activates interferon-stimulating gene (ISG) transcription by binding to the receptor and mediating its signal transduction. These genes determine the biological consequences of STAT1 signaling and mediate immune functions, inhibit cell proliferation, and induce apoptosis [30]. Moreover, a recent study showed that IFN-*α* can inhibit growth and induce apoptosis of HCC [31]. IFN-*γ* is mainly released by T cells that are recognized and activated by antigens [32], which can induce the expression of B7-H1 gene in lung cancer cells, bile duct cancer cells, head and neck cancers, and HCC through JAK/STAT1 pathway [32–35]. More mechanisms between HAIRGs and IFN pathway regulation in HCC need to be further explored.

Our study also found out different HAIRG signatures correlated with immune checkpoints expression in HCC, which include PD1, PDL1, PDL2, CTLA4, CD80, and CD86. The high expression of CTLA-4 on Tregs in HCC patients was negatively correlated with the cytolytic granzyme B produced by CD8+ T cells [36]. Meanwhile, both tumors and peritumoral cells (LSECs and HSCs) express the ligands PDL1 and PDL2, resulting in inactivation of CD8+ T cells that adhere to hepatic sinusoidal cells, which promotes immune tolerance. Besides, PDL1 expression in HCC also leads to follicular helper T cell failure and impairs the expression of cytokines and the help of B cells, therefore, promoting the development process to advanced tumor stage [36]. PD-1 may play an important function in promoting cancer development. PD-1 blocking combined with targeting mTOR pathway may enhance the antitumor curative effect in cancer [37, 38]. Erin et al. found that PD-1 blocking and IL-2 combined therapy could synergistically increase CD8+ T cell response [39]. Thus, there may be a mechanism between calcineurin inhibitors and PD-1 blockades that can serve as therapeutic sites for anti-HCC immunity [36]. However, due to the overexpression of miR-221, the expression of CD86 and CD40 on the surface of DCs downregulated, and miR-221 could delay the maturation of DCs in the microenvironment of HCC cells [40]. As the HAIRG signatures can predict immune checkpoint expression in HCC, our result also found that HAIRG signatures could predict the immunotherapy response in HCC patients. Therefore, our finding indicated the HAIRG signature could be a potential biomarker for the prediction of immunotherapy in HCC.

At last, we also explored the chemotherapeutic sensitivity of HCC patients based on HAIRG signature with traditional chemotherapeutic agents (cisplatin, AMG 706, gefitinib, and docetaxel). This study indicated that the high-risk patients might do better with cisplatin drug than the low-risk patients. However, when using the other drugs (AMG 706 and docetaxel), the low-risk patients did better as well as the high-risk patients. Cisplatin is a frequently used first-line chemotherapy drug for the treatment of liver cancer, ovarian cancer, small cell lung cancer, and other cancers [41]. Docetaxel has been widely utilized to relieve symptoms of breast, prostate, bladder, and ovarian cancers [42–44]. Furthermore, docetaxel has been recognized for its low toxicity and high efficacy in the treatment of liver cancer. Compared with previous studies [45–47], although had reported the hypoxic signatures, which included genes that PDSS1, SLC7A11, CDCA8, and angiogenesis-related immune signatures, such as BIRC5, KITLG, PGF, SPP1, and SHC1. These signatures of HCC had been reported can be used as potential biomarkers for diagnosis, prognosis, and recurrence of HCC. In our model, which had included more key tumor biological characteristics, included hypoxic, angiogenesis, and immune. More key tumor biological characteristics genes included in our model may construct a more accurate model for predicting the prognosis and treatment sensitivity in HCC. Our results also conducted immunotherapy response and chemotherapy sensitivity prediction in HCC. Therefore, we believe our model may be more novel and accurate. Therefore, our study firstly provides an effective and novel model to evaluate the chemosensitivity for HCC

## 5. Conclusion

In a word, our study defined a new prognostic model of HAIRGs in HCC patients. In the TCGA and ICGC cohorts, this model was shown to be independently associated with OS and can be considered a biomarker for prognosis prediction, clinical immunotherapy evaluation, and chemotherapy selection for HCC.

## Data Availability

The data and materials used to support the findings of this study are available from the corresponding author upon request.

## References

[1] Villanueva A. (2019). Hepatocellular Carcinoma. *The New England journal of medicine.*.

[2] Yang J. D., Hainaut P., Gores G. J., Amadou A., Plymoth A., Roberts L. R. (2019). A global view of hepatocellular carcinoma: trends, risk, prevention and management. *Nature reviews Gastroenterology & hepatology.*.

[3] Zhao Y., Huang X., Ding T. W., Gong Z. (2016). Enhanced angiogenesis, hypoxia and neutrophil recruitment during Myc-induced liver tumorigenesis in zebrafish. *Scientific Reports*.

[4] Weis S. M., Cheresh D. A. (2011). Tumor angiogenesis: molecular pathways and therapeutic targets. *Nature medicine.*.

[5] Yuen V. W., Wong C. C. (2020). Hypoxia-inducible factors and innate immunity in liver cancer. *The Journal of clinical investigation.*.

[6] Wen Q., Han T., Wang Z., Jiang S. (2020). Role and mechanism of programmed death-ligand 1 in hypoxia-induced liver cancer immune escape. *Oncology Letters*.

[7] Rooney M. S., Shukla S. A., Wu C. J., Getz G., Hacohen N. (2015). Molecular and genetic properties of tumors associated with local immune cytolytic activity. *Cell*.

[8] Jiang P., Gu S., Pan D. (2018). Signatures of T cell dysfunction and exclusion predict cancer immunotherapy response. *Nature medicine.*.

[9] Yang W., Soares J., Greninger P. (2013). Genomics of drug sensitivity in cancer (GDSC): a resource for therapeutic biomarker discovery in cancer cells. *Nucleic Acids Research*.

[10] Tellapuri S., Sutphin P. D., Beg M. S., Singal A. G., Kalva S. P. (2018). Staging systems of hepatocellular carcinoma: a review. *Indian journal of gastroenterology: official journal of the Indian Society of Gastroenterology.*.

[11] Claesson-Welsh L., Welsh M. (2013). VEGFA and tumour angiogenesis. *Journal of Internal Medicine*.

[12] Gao Q., Zhu H., Dong L. (2019). Integrated proteogenomic characterization of HBV-related hepatocellular carcinoma. *Cell*.

[13] Shin H. D., Park B. L., Cheong H. S., Yoon J. H., Kim Y. J., Lee H. S. (2007). SPP1 polymorphisms associated with HBV clearance and HCC occurrence. *International journal of epidemiology.*.

[14] Wang J., Hao F., Fei X., Chen Y. (2019). SPP1 functions as an enhancer of cell growth in hepatocellular carcinoma targeted by miR-181c. *American Journal of Translational Research*.

[15] Bandera Merchan B., Tinahones F. J., Macias-Gonzalez M. (2016). Commonalities in the association between PPARG and vitamin D related with obesity and carcinogenesis. *PPAR Research*.

[16] Zhang S., Jiang J., Chen Z. (2018). Relationship of <em>PPARG</em>, <em>PPARGC1A</em>, and <em>PPARGC1B</em> polymorphisms with susceptibility to hepatocellular carcinoma in an eastern Chinese Han population. *Oncotargets and Therapy*.

[17] Campbell M. J., Carlberg C., Koeffler H. P. (2008). A role for the PPAR in cancer therapy. *PPAR research.*.

[18] Park C. S., Eom D. W., Ahn Y., Jang H. J., Hwang S., Lee S. G. (2019). Can heme oxygenase-1 be a prognostic factor in patients with hepatocellular carcinoma?. *Medicine*.

[19] Chiang S. K., Chen S. E., Chang L. C. (2019). A dual role of heme oxygenase-1 in cancer cells. *International Journal of Molecular Sciences*.

[20] Shi W., Feng L., Dong S. (2020). FBXL6 governs c-MYC to promote hepatocellular carcinoma through ubiquitination and stabilization of HSP90AA1. *CCS.*.

[21] Xu Q., Tu J., Dou C. (2017). HSP90 promotes cell glycolysis, proliferation and inhibits apoptosis by regulating PKM2 abundance via Thr-328 phosphorylation in hepatocellular carcinoma. *Molecular cancer.*.

[22] Kang G. H., Lee E. J., Jang K. T. (2010). Expression of HSP90 in gastrointestinal stromal tumours and mesenchymal tumours. *Histopathology*.

[23] Li Y., Zhang T., Schwartz S. J., Sun D. (2011). Sulforaphane potentiates the efficacy of 17-allylamino 17-demethoxygeldanamycin against pancreatic cancer through enhanced abrogation of Hsp 90 chaperone function. *Nutrition and Cancer*.

[24] Soroka J., Wandinger S. K., Mäusbacher N. (2012). Conformational switching of the molecular chaperone Hsp90 via regulated phosphorylation. *Molecular Cell*.

[25] Shan-Shan Jiang D.-S. W., Wang Q.-J., Pan K. (2014). Galectin-3 is associated with a poor prognosis in primary hepatocellular carcinoma. *Journal of Translational Medicine*.

[26] Blanquart C., Gueugnon F., Nguyen J. M. (2012). CCL2, galectin-3, and SMRP combination improves the diagnosis of mesothelioma in pleural effusions. *Journal of Thoracic Oncology: Official Publication of the International Association for the Study of Lung Cancer*.

[27] Matsuda Y., Yamagiwa Y., Fukushima K., Ueno Y., Shimosegawa T. (2008). Expression of galectin-3 involved in prognosis of patients with hepatocellular carcinoma. *Hepatology research: the official journal of the Japan Society of Hepatology.*.

[28] Mayoral M. A., Mayoral C., Meneses A. (2008). Identification of galectin-3 and mucin-type O-glycans in breast cancer and its metastasis to brain. *Cancer investigation.*.

[29] Li T., Dong Z. R., Guo Z. Y. (2013). Aspirin enhances IFN-*α*-induced growth inhibition and apoptosis of hepatocellular carcinoma via JAK1/STAT1 pathway. *Cancer Gene Therapy*.

[30] Khodarev N. N., Roizman B., Weichselbaum R. R. (2012). Molecular pathways: interferon/stat1 pathway: role in the tumor resistance to genotoxic stress and aggressive growth. *Clinical cancer research: an official journal of the American Association for Cancer Research.*.

[31] Herzer K., Hofmann T. G., Teufel A. (2009). IFN-alpha-induced apoptosis in hepatocellular carcinoma involves promyelocytic leukemia protein and TRAIL independently of p 53. *Cancer research.*.

[32] Li N., Wang J., Zhang N. (2018). Cross-talk between TNF-*α* and IFN-*γ* signaling in induction of B7-H1 expression in hepatocellular carcinoma cells. *Cancer immunology, immunotherapy: CII.*.

[33] Concha-Benavente F., Srivastava R. M., Trivedi S. (2016). Identification of the cell-intrinsic and -extrinsic pathways downstream of EGFR and IFN*γ* that induce PD-L1 expression in head and neck cancer. *Cancer Research*.

[34] Lee S. J., Jang B. C., Lee S. W. (2006). Interferon regulatory factor-1 is prerequisite to the constitutive expression and IFN-gamma-induced upregulation of B7-H1 (CD274). *FEBS letters.*.

[35] Gong A. Y., Zhou R., Hu G. (2009). MicroRNA-513 regulates B7-H1 translation and is involved in IFN-gamma-induced B7-H1 expression in cholangiocytes. *Journal of immunology.*.

[36] Zongyi Y., Xiaowu L. (2020). Immunotherapy for hepatocellular carcinoma. *Cancer letters.*.

[37] Munker S., De Toni E. N. (2018). Use of checkpoint inhibitors in liver transplant recipients. *United European gastroenterology journal.*.

[38] Li H., Li X., Liu S. (2017). Programmed cell death-1 (PD-1) checkpoint blockade in combination with a mammalian target of rapamycin inhibitor restrains hepatocellular carcinoma growth induced by hepatoma cell-intrinsic PD-1. *Hepatology*.

[39] West E. E., Jin H. T., Rasheed A.-U. (2013). PD-L1 blockade synergizes with IL-2 therapy in reinvigorating exhausted T cells. Research article.

[40] Fu Y., Li F., Zhang P. (2019). Myrothecine A modulates the proliferation of HCC cells and the maturation of dendritic cells through downregulating miR-221. *International Immunopharmacology*.

[41] Wang Y., Liu Y., Liu Y. (2015). A polymeric prodrug of cisplatin based on pullulan for the targeted therapy against hepatocellular carcinoma. *International Journal of Pharmaceutics*.

[42] Albany C., Sonpavde G. (2015). Docetaxel for the treatment of bladder cancer. *Expert opinion on investigational drugs.*.

[43] Belz J., Castilla-Ojo N., Sridhar S., Kumar R. (2017). Radiosensitizing silica nanoparticles encapsulating docetaxel for treatment of prostate cancer. *Methods in molecular biology.*.

[44] Seguin C., Kovacevich N., Voutsadakis I. A. (2017). Docetaxel-associated myalgia-arthralgia syndrome in patients with breast cancer. *Breast Cancer*.

[45] Mo Z., Liu D., Rong D., Zhang S. (2021). Hypoxic characteristic in the immunosuppressive microenvironment of hepatocellular carcinoma. *Frontiers in Immunology*.

[46] Zhang B., Tang B., Gao J., Li J. (2020). A hypoxia-related signature for clinically predicting diagnosis, prognosis and immune microenvironment of hepatocellular carcinoma patients. *Journal of Translational Medicine*.

[47] Yang Y., Wu G., Li Q. (2021). Angiogenesis-related immune signatures correlate with prognosis, tumor microenvironment, and therapeutic sensitivity in hepatocellular carcinoma. *Frontiers in molecular biosciences.*.

